# A Smart Power Electronic Multiconverter for the Residential Sector

**DOI:** 10.3390/s17061217

**Published:** 2017-05-26

**Authors:** Miguel Angel Guerrero-Martinez, Maria Isabel Milanes-Montero, Fermin Barrero-Gonzalez, Victor Manuel Miñambres-Marcos, Enrique Romero-Cadaval, Eva Gonzalez-Romera

**Affiliations:** Power Electrical and Electronic Systems Research Group, Escuela de Ingenierías Industriales, Universidad de Extremadura, Avda. de Elvas, s/n, Badajoz 06006, Spain; milanes@unex.es (M.I.M.-M.); fbarrero@unex.es (F.B.-G.); vminmar@unex.es (V.M.M.-M.); eromero@unex.es (E.R.-C.); evagzlez@unex.es (E.G.-R.)

**Keywords:** smart grid, energy management system, smart community, supercapacitor, hybrid energy storage system, multiconverter

## Abstract

The future of the grid includes distributed generation and smart grid technologies. Demand Side Management (DSM) systems will also be essential to achieve a high level of reliability and robustness in power systems. To do that, expanding the Advanced Metering Infrastructure (AMI) and Energy Management Systems (EMS) are necessary. The trend direction is towards the creation of energy resource hubs, such as the smart community concept. This paper presents a smart multiconverter system for residential/housing sector with a Hybrid Energy Storage System (HESS) consisting of supercapacitor and battery, and with local photovoltaic (PV) energy source integration. The device works as a distributed energy unit located in each house of the community, receiving active power set-points provided by a smart community EMS. This central EMS is responsible for managing the active energy flows between the electricity grid, renewable energy sources, storage equipment and loads existing in the community. The proposed multiconverter is responsible for complying with the reference active power set-points with proper power quality; guaranteeing that the local PV modules operate with a Maximum Power Point Tracking (MPPT) algorithm; and extending the lifetime of the battery thanks to a cooperative operation of the HESS. A simulation model has been developed in order to show the detailed operation of the system. Finally, a prototype of the multiconverter platform has been implemented and some experimental tests have been carried out to validate it.

## 1. Introduction

Electricity networks have become the backbone of the development of society during the twentieth century, mainly because the world energy demand increases every year. A growth by more than one-third is expected until 2035 [1]; fossil fuels remain dominant to satisfy this demand, however, according to the International Energy Agency (IEA), a trend change is observed with the growth of low carbon technologies [2], so renewable energies could reach 25% of the global energy mix in 2018, even more with the Hosting Capacity (HC) increase [3]. A general concern for energy efficiency has led to new challenges such as making better use of renewable energy, reducing greenhouse gas emissions, improving energy management, matching the consumption and energy generation, incorporating energy storage into electrical grids, avoiding the transport of the excess energy, and developing a more efficient and reliable electrical grid to ensure a safe power supply. To reach all these objectives the smart grids concept was born. A smart grid is an advanced electrical grid with the intelligence and capacity to perform all these functions. There is no unique definition for smart grids; this term is a relatively new in the power system sector. The European Technology Platform (ETP) developed in 2006 the concept of smart grids giving a brief definition: “*electricity network that can intelligently integrate the actions of all users connected to it—generators, consumers and those that do both—in order to efficiently deliver sustainable, economic and secure electricity supplies*” [4].

The smart grid is critical in the integration of renewables in the distribution network, as well as for proper implementation of Distributed Generation (DG) [5,6,7,8,9,10]. Other factor to consider in developing the smart grid is significant energy losses that occur in power networks. In transmission network energy losses are estimated about 2–4%, while in distribution networks, it is estimated that losses are 4–9% [11]. Around 10% of the energy produced is wasted because of a lack of proper energy management that increases the costs of electricity supply. For example, in the United States more than half of the electricity produced is lost as a result of the lack of coordination in the management of the generation, transmission and distribution networks [12].

The operation of the new electrical grids is changing and the electrical systems must be adapted to the expectations of society. The new role of future grids includes to change the traditional inflexible passive approach to grid operation, becoming to active electrical systems that incorporate new advanced power devices that can respond the needs of electricity customers. The active approach to grid operation permits the interaction between all the elements of the distribution network.

The smart grid implementation requires power converters with general purposes, Advanced Metering Infrastructure (AMI) and Information and Communication Technology (ICT) to obtain power consumption profiles and electrical parameters [13]. The trend goes towards the creation of energy resource hubs, such as the Smart Community concept. The converters in these intelligent communities allow developing future functions that make the grid smart. Some of the future capabilities keep relation with the bulky storage, spinning reserve and ancillary services, voltage support [14], reduce the electricity bill of the residential customers [15], management cost minimization and congestion relief [16], fulfilling the demand of various shared facilities [17], minimizing total cost and power forecasts [18,19], resource adequacy [20], and improving the efficiency [21].

The new topology of converters incorporates energy storage, allowing smoothing of peak power to power shift peak demand [22], improving efficiency [23], constant and stable output power [24], system reliability [25], suppress PV power fluctuation and renewables integration [26,27].

Following these lines of research, this paper aims to provide the design and experimental validation of a power electronic multiconverter system for smart grid applications. Specifically, the multiconverter platform is tested for a residential-scale application, which integrates distributed PV modules and a hybrid storage (battery and supercapacitor) system. This device would be installed in homes of a smart community. A central EMS manages the active energy flow between the grid, the distributed multiconverters and the loads existing in the community and sends individual active power set-points to each multiconverter system. Thus, each equipment is responsible for demanding or injecting into the grid the reference active power established by the smart community EMS and, at the same time, guaranteeing that the local PV modules operate according to a MPPT algorithm to maximize the power extraction under all conditions. The storage system of the multiconverter behaves as a local energy buffer that ensures that the active power balance in each house of the community is met and operates according to a collaborative strategy that allow extending the lifetime of the storage elements. The main contributions of the paper are:

- the proposal of a general-purpose robust and compact multiconverter system, designed for smart grid applications. It includes one renewable energy source and up to two energy storage elements which can be controlled independently or cooperatively. A 3 kVA three-phase multiconverter laboratory prototype has been built for experimental tests. The design of the measurement stage is described in detail.
- the application of the multiconverter system to a local energy unit installed in homes of a smart community operating according to the active power set-points received from the community EMS.
- the validation by simulation and experimental tests of the proper operation of the multiconverter in the proposed residential sector application.
- the performance of this multiconverter presents several improvements compared with traditional converters, such as:
  - tracking an active power set-point, demanding or injecting into the community grid sinusoidal and balanced currents although the grid voltage is distorted or unbalanced,
  - energy storage based on a hybrid configuration, composed by batteries and supercapacitors, managed cooperatively in order to improve the efficiency and extend the lifetime of the storage elements,
  - integration into a common platform equipped with real-time communication systems of a local power unit capable of adapting MPPT PV generation, hybrid storage and consumption following instantaneous set-points sent by the EMS of the community.

The advantages for the citizens of this smart community are clear. From an economical point of view, they can reduce the maximum power contracted with the utility (i.e., getting a lower electric bill) and, if the global community management allows selling the surplus energy to the utility company, the benefits could be shared among all the houses in the community. Besides, the investment in the storage system is favored due to the use of a hybrid system, which allows increasing lifetime of the battery, reducing costs of equipment replacement or renewal. At the same time, the citizens contribute to help the environment, increasing the amount of electricity consumption produced from renewable sources and extending the lifetime of the storage elements.

The paper is structured as follows: in Section 2 the topology chosen for the multiconverter system is presented. Section 3 shows the designed boards. The control algorithms are explained in Section 4. Section 5 is devoted to validate, by simulation, how the system is able to fulfill the active power set-points and guarantee a MPPT tracking operation of the PV modules while managing properly the HESS. Once the simulation tests are carried out, in Section 6 the prototype is presented and validated experimentally. Finally, the conclusions are summarized.

## 2. Topology of the Multiconverter System

**Figure 1:**
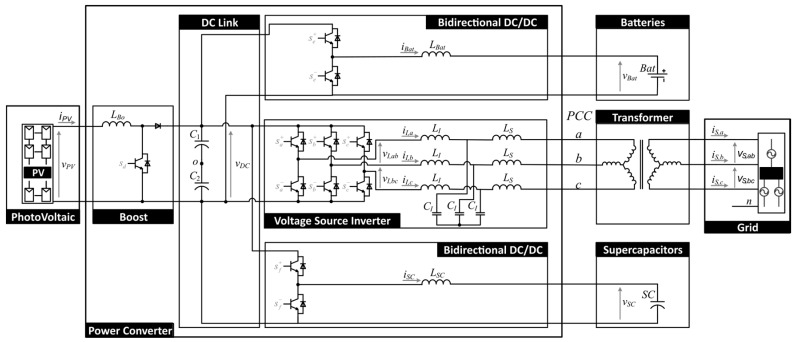
Scheme of the proposed multiconverter topology.

The multiconverter topology consists of four converters with a common DC link (Figure 1): a boost stage, two bidirectional DC/DC converters, and a traditional 3-phase power grid-connected inverter. This general-purpose power converter topology allows to include renewable energy sources, storage systems, and other necessary devices to fulfill the smart grid goals.

To test this multiconverter system, an application for the residential/housing sector is proposed in this paper: a smart community with a renewable energy source and energy storage elements. Thus, as displayed in Figure 1, the platform includes some photovoltaic modules, connected to the DC link through the unidirectional boost converter and also two energy storage elements, a battery module and a supercapacitor, connected to the DC link by using bidirectional DC/DC converters. It allows to test the platform with a hybrid storage system, formed by batteries and supercapacitors.

A smart community EMS receives from the multiconverter installed in each house the instantaneous energy generation and consumption, and the State of Charge (SOC) of the battery. It provides an active power set-point to each multiconverter, aiming to optimize globally the energy billing of the whole community, minimizing losses and getting the highest efficiency of the generation and storage units available in the community. The HESS operates with a cooperative strategy, since the supercapacitors covers peaks, transients and fast power fluctuations in the active power set-point. It helps to avoid battery stress due to high charging currents and micro-charging cycles, increasing battery lifetime. In this way, the battery only has to cover the average active power set-point.

On the other hand, the power grid inverter is responsible for demanding or injecting into the grid the active power established as reference by the smart community EMS. The control algorithm of this inverter guarantees that the multiconverter operates with high power quality, since the demanded or injected current is sinusoidal and balanced, even in case of distorted or unbalanced grid voltages. The inverter connected to the grid, as shown in Figure 1, using a LCL filter.

## 3. Control System: Control Board and Measurement Stage

**Figure 2:**
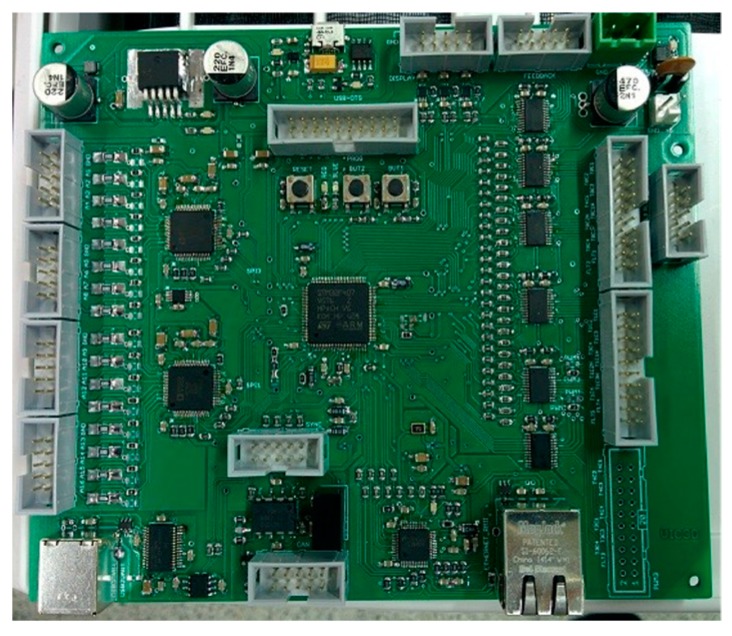
Control board based on the STM32F407 microcontroller.

The control system has been implemented in a control board based on the STM32F407 microcontroller by STMicroelectronics (Geneva, Switzerland, Figure 2). This embedded microcontroller processor, which is based on the Cortex-M4F ARM Reduced Instruction Set Computer (RISC, Cambridge, UK) type 32-bit high-performance core 1 MB of flash memory and 192 Kb of RAM, operates at a maximum frequency of 168 MHz. The core Cortex-M4F has a Floating Point Unit (FPU) that supports data processing instructions ARM, and four DMA controllers (two for general purpose, one USB, one Ethernet). It also implements a complete set of DSP instructions, a memory protection unit (MPU) to improve the security and a nested vectored interrupt controller (NVIC) (configurable external interrupts, priority bit, prioritization of interruptions). For the development of the control software, a GCC compiler is used, and an IDE CooCox, which is a free development environment for microcontrollers with core Cortex-M series manufactured by ARM. The board has Ethernet communication for connection to the control network (Smart Community network for EMS-multiconverters communication).

The measurement stage has been implemented by Hall Effect transducers, with galvanic isolation, LA 55P/SP1 (LEM) for measuring DC and AC currents and LV 25-P for measuring DC and AC voltages. The characteristics of these sensors are collected in Table 1. This type of sensor works with high precision, good linearity and low common mode disturbance.

**Figure 3:**
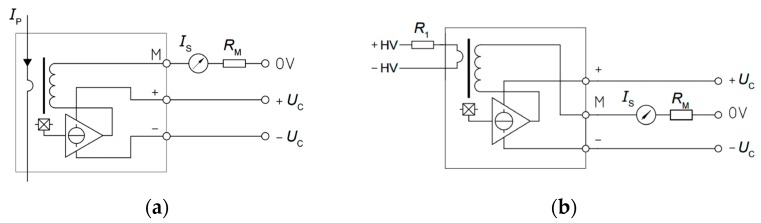
Schematic circuits of the AC and DC voltage and current sensors: (**a**) Current transducers; (**b**) Voltage transducers.

In the current transducers LA 55P/SP1 (Figure 3a), the magnetic flux created by the primary current *I_P_* is balanced by a complementary flux produced by the current through the winding of the secondary. This Hall Effect device is associated with an electronic circuit to generate the compensation current in the secondary for an accurate representation of the primary current. An additional advantage for this configuration is that the secondary coil works as a current transformer, extending the bandwidth and reducing the time response of the transducer.

A Hall Effect closed-loop sensor is sometimes called a ‘Zero-Flux’ sensor, because its Hall-Effect sensor feeds back an opposite current into a secondary coil, wound on the magnetic core, to carry to zero the flux produced in the magnetic core by the primary current. For this reason, the meter includes a compensation circuit that tries to cancel the magnetic flux produced by the current to be measured. In these sensors the current is indirectly measured through the voltage on resistance *R*_M_. The internal error amplifier measures the net flow through the ferrite by using a Hall element. The output of the amplifier is connected to the output stage which injects a current, canceling the flux produced by the current.

The main difference between voltage and current transducers is the addition of an internal primary coil with a large number of windings, allowing the sensor to set the ampere-turns needed to measure the small current flowing in the primary. The operating principle of the voltage transducer LV 25-P (Figure 3b) is based on a small current limited by a resistor in series with the voltage to be measured, driven through the primary coil. The magnetic flux created by the primary current *I*_P_ is balanced by a complementary flux produced by a current through the secondary winding. As the above transducer, it consists of a Hall Effect device and an associated electronic circuit to perform the compensation. The primary resistance (*R*_1_) can be included or not in the transducer.

**Figure 4:**
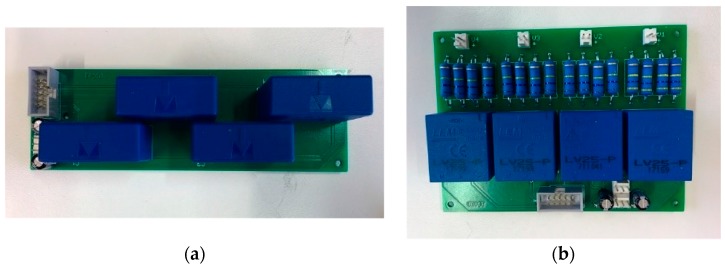
Sensor boards: (**a**) current sensor board used in the experimental setup to measure the current control variables; (**b**) voltage sensor board used in the experimental setup to measure the voltage control variables.

To complete the control platform two sensors boards with four LA 55P/SP1 current transducers (Figure 4a) and two sensor boards with two LV 25-P voltage transducers (Figure 4b) have been developed.

## 4. Control Algorithm

**Figure 5:**
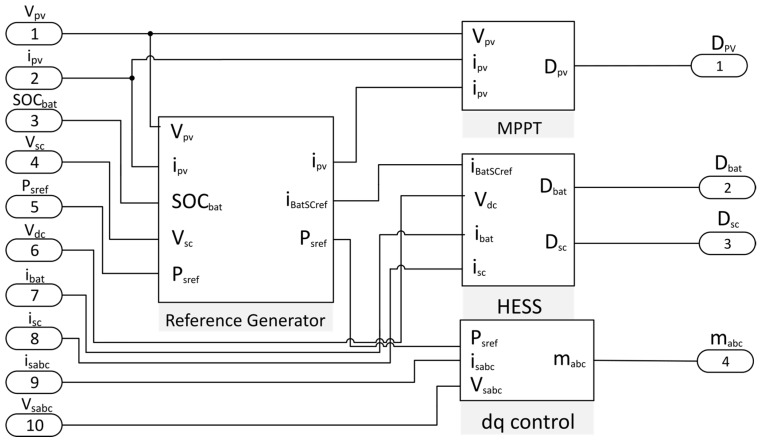
Control strategies for the multiconverter system.

The global control strategy developed to manage the multiconverter is displayed in Figure 5. This control algorithm is responsible for generating the duty cycles of the converters. It is formed by a main block, the reference generator, and three additional blocks: the MPPT, the HESS and the *d-q* control block for the grid-connected inverter.

As can be seen from Figure 5, the control strategy of the reference generator measures the PV voltage, the PV current, the SOC of the battery and the supercapacitor voltage. It also receives as input the reference active power, P_Sref_, delivered by the EMS of the smart community. The outputs of this block are the reference current for the MPPT tracker, the reference current for the HESS block, and the reference power for the grid-connected inverter [28]. In the following sections a brief explanation of the additional blocks which make up this control stage is provided.

### 4.1. MPPT Control Logic

The reference generation of the MPPT is based on the incremental conductance theory [29,30]. This method observes the P-V curve maximum power point. The slope of the curve of the PV module is zero at the Maximum Power Point (MPP), positive to the left of the MPP and negative to the right of the MPP.

(1) $$\frac{dp_{PV}}{dv_{PV}}=\frac{d(i_{PV}v_{PV})}{dv_{PV}}=i_{PV}+i_{PV}\frac{di_{PV}}{dv_{PV}}$$

The MPP is achieved by comparing the instantaneous conductance (I/V) with the incremental conductance (ΔI/ΔV). The reference voltage of the PV module is the converter maximum power point voltage, increasing when the operation point is on the left of the MPP and decreasing when is on the right side.

The PV module reference current generation is obtained by using an integral controller or limiting it, according to the reference generator controller. Then, using a proportional-integral (PI) controller the duty cycle of the converter is generated.

**Figure 6:**
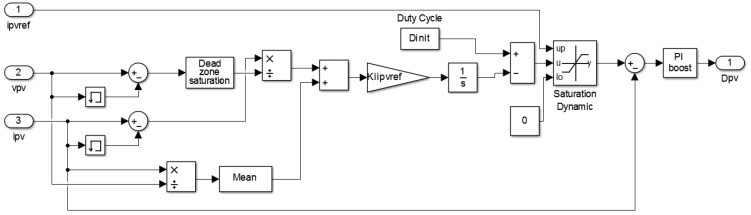
MPPT control block scheme.

The inputs of this block, displayed in Figure 6, are the voltage, current and reference current of the photovoltaic panel, and it generates the duty cycle of the boost converter as output, D_PV_.

### 4.2. HESS Control

In the HESS control stage a *V_DC_* regulation block, by using a PI controller, calculates the HESS reference current which maintains the DC bus voltage at its reference value, V_DCref_. Once the global HESS reference current is calculated, the Hybrid Logic block distributes this current between the battery and the supercapacitor. In this paper, the most used sharing control strategy for parallel active hybrid topologies [31] has been selected: since the goal is that the battery covers the average active power set-point, delivering the peaks and power fluctuations to the supercapacitor, a first order Low Past Filter (LPF) with time constant τ is employed [32,33,34,35,36]. This simple sharing strategy guarantees that the dynamic stress and losses of the battery are reduced, increasing its lifetime. Thus, the reference is divided into the reference battery current, responsible for the low frequency components, and the reference supercapacitor current, that handles the high ones.

The battery and supercapacitor control blocks include PI current controllers that ensure that each storage current follow its corresponding reference current with the minimum error.

**Figure 7:**
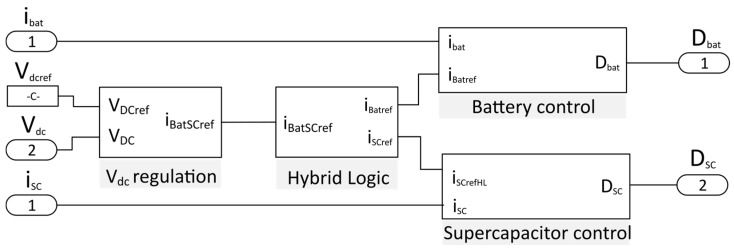
HESS control diagram.

The inputs of the HESS control, shown in Figure 7, are the battery current, the supercapacitor current, the DC link voltage and the reference DC link voltage. The outputs are the duty cycle of the bidirectional converters D_bat_ and D_SC_ responsible for the energy storage control.

### 4.3. d-q Inverter Control

The inverter control is based on the direct–quadrature theory, where the phase-to-neutral mains voltage, v_sabc_ and currents, i_sabc_, are transformed into the *d-q* voltages, v_dq_, and currents, i_dq_, [28]. The Phase Locked Loop (PLL) is synchronized to the positive-sequence fundamental component of the source voltage [37,38]. The inverter control signals are obtained in the *d-q* frame by a feed-forward scheme that decouples the terms *d-q* and two PI controllers [28]:

(2) $$m_{d}=v_{d}-RI_{d}+\omega LI_{q}+L\frac{di_{d}}{di_{t}},$$

(3) $$m_{q}=v_{q}-\omega LI_{d}+RI_{q}R+L\frac{di_{q}}{di_{t}},$$

where *L* is the inductance of the filter inductor and *R* is its internal resistance. The inverter modulation indexes m_abc_ are finally obtained from the *d-q* to *a-b-c* transformation.

The i_dqref_ block includes a Balanced Sinusoidal Source Current (BSSC) [39] control strategy, calculating an inverter reference current in phase with the positive-sequence fundamental grid voltage, ensuring sinusoidal and balance inverter currents although the grid voltage is distorted or unbalanced.

**Figure 8:**
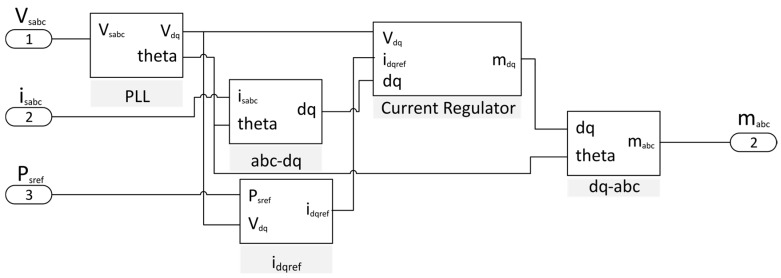
*d-q* control diagram.

The inputs of the inverter control algorithm, displayed in Figure 8, are the source voltages and currents and the reference active power provided as a set-point by the smart community EMS. The output is the duty cycle of the inverter, m_abc_.

## 5. Simulation Analysis

**Figure 9:**
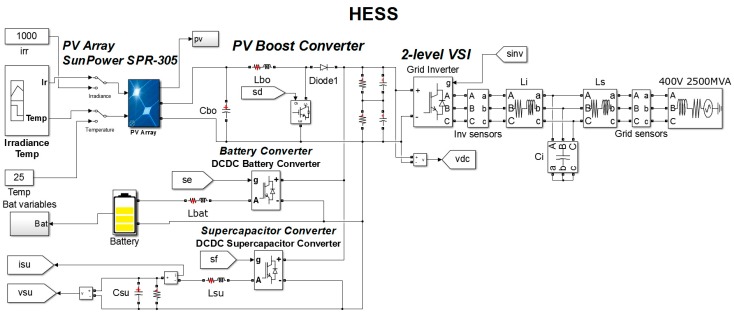
System simulation models using MATLAB/Simulink.

The system shown in Figure 1 has been simulated by employing the libraries components of Simscape Power Systems™ MATLAB/Simulink (Hydro-Québec, Montreal, QC, Canada) (Figure 9). The control strategy is implemented using the control algorithm explained in the previous section.

The main simulation parameters values are summarized in Table 2. The passive elements of this topology, collected in Table 3, are designed to provide voltage and current within allowable margins [40]. The input variables are the irradiance, *G*, the temperature *T*, and the RMS grid voltage. The user interface variable is the reference active power set-point, P_ref_.

Some simulation tests has been proposed in order to demonstrate that the multicoverter achieves the three proposed objectives: fulfilling the active power set-points provided by the EMS, MPPT of PV modules and cooperative operation of the HESS.

**Figure 10:**
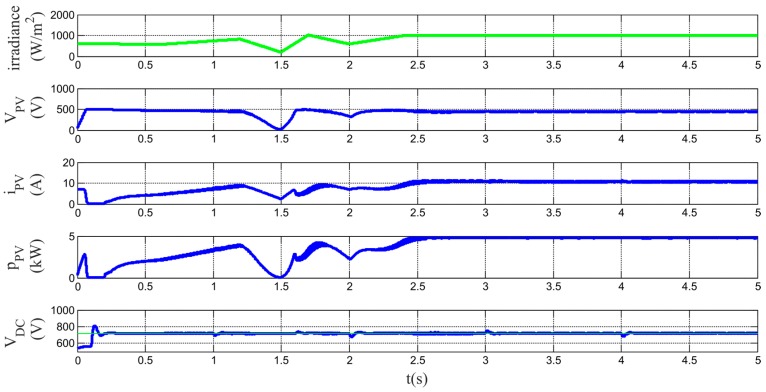
Simulation results. From top to bottom: irradiance variation pattern, PV module voltage, PV module current, PV module power and DC bus link voltage. Green lines are used for references and blue lines are for measurements.

Figure 10 shows the proper MPPT operation during the analyzed interval and how the HESS maintains the DC bus voltage at the reference value.

In order to show the active power set-points tracking capability of the multiconverter, a simulation test is proposed with the following pattern: from t = 0 to t = 1 s, P_Sref_ = 0 kW; from t = 1 s to t = 2 s, P_Sref_ = 2.5 kW; from t = 2 s to t = 3 s, P_Sref_ = 5 kW; from t = 3 s to t = 4 s, P_Sref_ = 2.5 kW, from t > 4 P_Sref_ = 5 kW.

**Figure 11:**
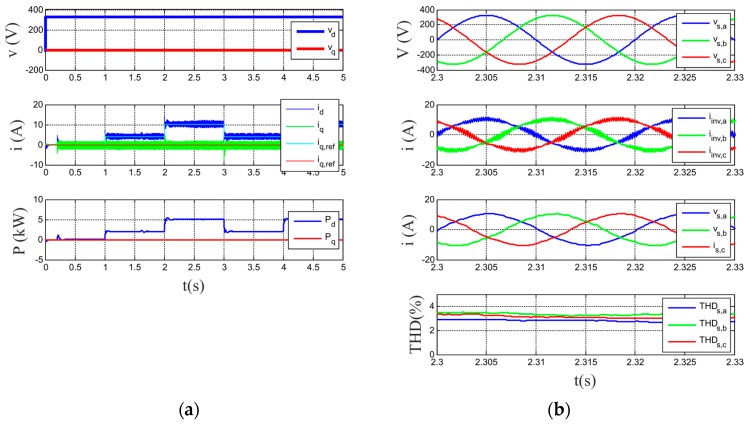
Simulation results. (**a**) from top to bottom: *d-q* voltage components, *d-q* current components and power injected by the system. (**b**) from top to bottom: source voltages, inverter currents, currents injected into the grid and THD of these grid currents.

**Figure 12:**
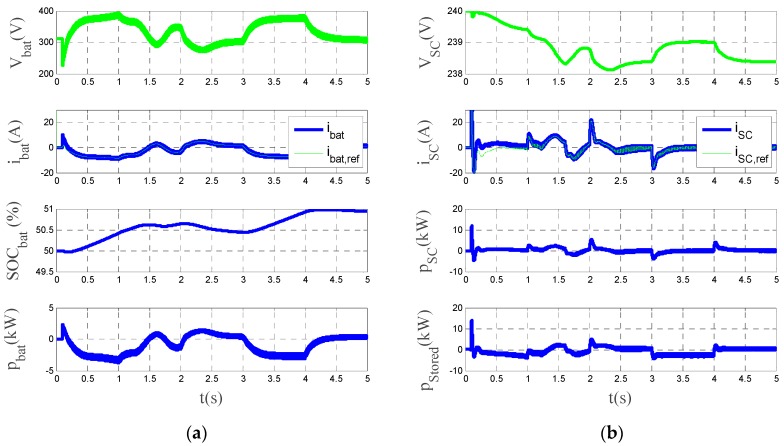
Simulation results showing the operation of the HESS. (**a**) from top to bottom: battery voltage, battery current, SOC of the battery and battery power. (**b**) from top to bottom: supercapacitor voltage, supercapacitor current, supercapacitor power and total HESS power.

Figure 11a shows the *d-q* components of the grid voltage and grid current and the power injected into the grid. Figure 11b displays a zoom window interval is shown, displaying the three-phase phase-to-neutral source voltages, inverter currents and source currents after the LCL filters. Finally, the Total Harmonic Distortion of the source currents are shown. As can be seen in the figure, the power injected by the system follows the established power set-points for the entire time interval. Source currents are balanced and sinusoidal with a Total Harmonic Distortion (THD) remaining below 5%, as it is regulated by standards [41,42]. Finally, Figure 12 shows the collaborative operation performed by the HESS. The hybrid system is responsible for sharing the reference HESS current between the battery and the supercapacitor, so that there are no current peaks in the battery and avoiding big changes of voltage. One can notice from this figure that the battery has a proper behavior during charging and discharging, avoiding current peaks, and the supercapacitor works to absorb the current peaks and so improving the lifetime of the battery.

## 6. Experimental Results

**Figure 13:**
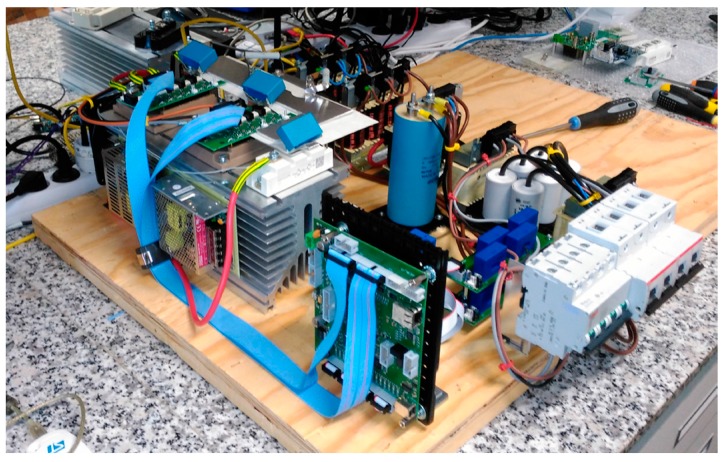
Multiconverter prototype.

The experimental prototype to validate the operation of the multiconverter is shown in Figure 13 and the parameters are summarized in the Table 4. This prototype contains the same elements as the topology shown in Figure 1.

**Figure 14:**
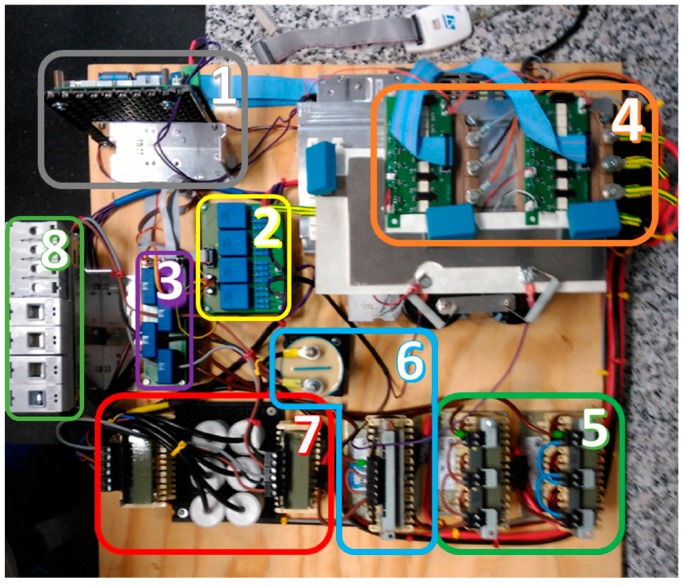
Laboratory prototype components: (1) control board; (2) voltage sensors; (3) current sensors; (4) intelligent power modules (IPM); (5) filters of bidirectional converter; (6) passive component of boost converter; (7) output grid filters; (8) output protections.

**Figure 15:**
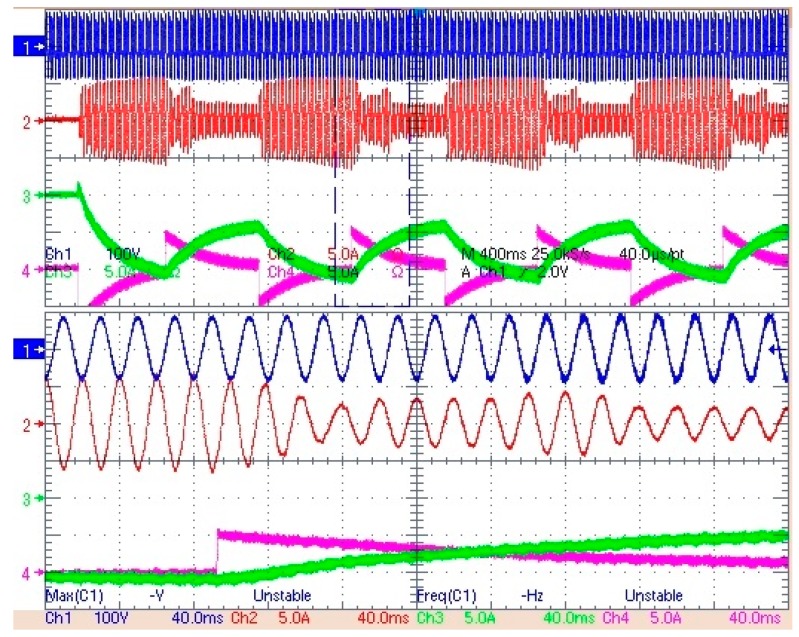
Experimental results. From top to bottom: source voltage (in blue), source current (in red), battery current (in green) and supercapacitor current (in magenta).

The main components of the multiconverter prototype are enumerated in Figure 14. It has a first connection stage with AC and DC protecting, and then, a stage of currents and voltage measurements. Below, it is the filtering stage: LCL filters for the three phase inverter, the capacitor and the coil of the lifting stage, and two coils for bidirectional converters. Two Fuji Intelligent Power Modules (IPM) share a common DC bus. Finally, it is the control unit, which manages the measurements, control signals and alarms. The HP E4351B Solar Array Simulator has been used to emulate the PV panels and the specifications of the Li-ion battery and supercapacitor are collected in Table 4. Experimental tests have been carried out to validate the control system and the multiconverter performance with a rated power of 3 kW. First, an experimental test has been proposed to show the behavior of the system when the smart community EMS sends reference active power set-points changing from −3 kW to −1.7 kW every 500 ms. Since *P_Sref_* is negative, it means that the HESS has to charge. The results are displayed in Figure 15. This figure shows, at the top, the behavior of the multiconverter during a test interval: in blue the source voltage, in red the source current, in green the battery current and in magenta the supercapacitor current. One can notice that when the change in the set-point occurs, the supercapacitor comes into operation taking charge of the change step, progressively leaving the battery to operate. These results demonstrate that, indeed, the collaboration strategy for the HESS works correctly. At the bottom of the figure, a zoom of the same variables is displayed. As P_Sref_ is negative, the source current is sinusoidal, but opposite in phase with the source voltage.

**Figure 16:**
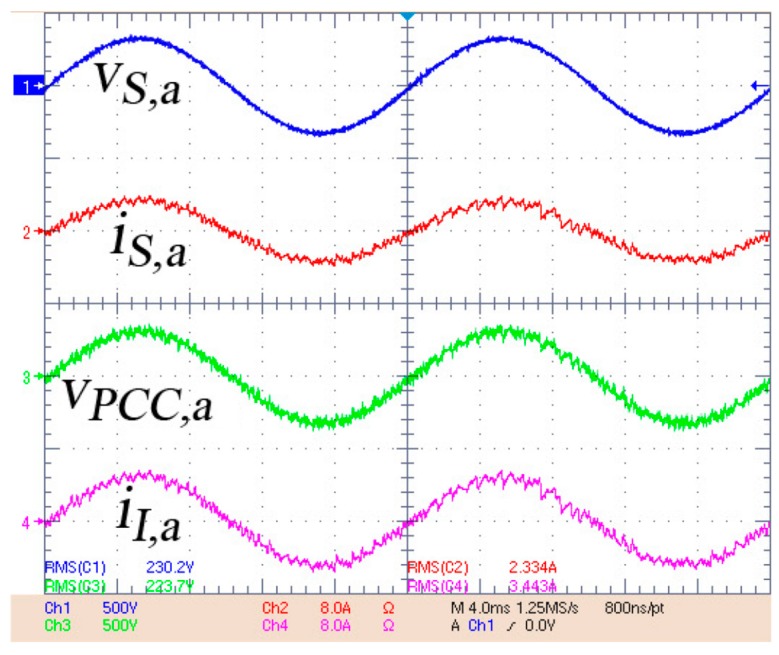
Experimental results injecting active power into the grid. From top to bottom: source voltage (in blue), source current (in red), AC inverter voltage (PCC voltage) (in green) and inverter current (in magenta).

In Figure 16 the system response is shown when a positive active power reference P_Sref_ = 2 kW is provided by the smart community EMS. It means that the multiconverter is injecting active power into the community grid and the HESS is discharging. In this figure it is displayed, from top to bottom: the source voltage, the source current, the AC inverter voltage (PCC voltage) and the inverter current. The source current is sinusoidal and in phase with the positive-sequence fundamental source voltage, so no harmonic distortion is injected into the grid and the multiconverter operates with unity displacement power factor. These results demonstrate that the traking of the active power set-point is achieved by the multiconverter with high power quality.

## 7. Conclusions

This paper presents a power electronic converter with a multiconverter topology provided with a renewable energy source and up to two energy storage elements, which allows managing the energy available in houses connected to a smart community grid. This device permits, within the smart grid scenario, developing new tasks for local energy units installed in homes with small PV generation and hybrid storage elements.

A bidirectional active power flow control between the multiconverter and the community grid has been implemented, following the active power set-points provided by the smart community EMS. This central element receives from each house the instantaneous energy generation and consumption, and the SOC of the battery. It provides to each multiconverter unit an active power set-point, aiming to globally optimize the energy billing of the whole community, minimizing losses and getting the highest efficiency of the generation and storage units available in the community

After the simulation and experimental tests, it can be concluded that this multiconverter presents several improvements compared with traditional converters. It attains simultaneously: to track an active power set-point with high power quality; operate PV modules with MPPT algorithm; and managing energy storage based on a hybrid configuration, composed by batteries and supercapacitors, in order to improve the efficiency and lifetime of the storage elements.

## Figures and Tables

**Table 1:** Characteristics of AC and DC voltage and current sensors.

Current Transducers	Specification	Voltage Transducers
0 ... ±100 A	Measuring range	0 ... ±14 mA (10 mA/500 V)
25 mA	Secondary nominal current rms	25 mA
±12 ... 15 V	Supply voltage	±15 V (±5%)
200 kHz	Frequency bandwith	200 kHz
2.5 kV rms	Isolation	2.5 kV rms
1:2000	Conversion ratio	2500:1000
±0.9%	Accuracy	±0.9%
−40 °C ... 85 °C	Operating temperature	0 °C ... 70 °C

**Table 2:** Main parameters values used in the converter simulation model.

Parameter	Value
PV nominal power	5 kW
DC reference voltage	720 V
Battery storage capacity	3 kWh
Nominal battery voltage	48 V
Supercapacitor storage capacity	0.3 kWh
Supercapacitor equivalent circuit capacitance	37.5 F
Low pass filter time constant	0.2 s
PWM switching frequency	5 kHz

**Table 3:** Values of the filter parameters used in the converter.

Parameter	Value
*C*_1_, *C*_2_	2.2 mF
*L*_Bo_	10 mH
*L*_Bat_	20 mH
*L*_SC_	20 mH
*L_I_*, *C_I_*, *L_S_*	5 mH, 10 µF, 5 mH

**Table 4:** Experimental parameters.

PV Panels	**HP E4351B Solar Array Simulator**
Power Electronics	**Fuji IGBT-IPM 6MBP50RA120**
Battery	**Li-ion batteries BMZ energy storage 6.74 kWh (Li-Ion NMC)** Nominal voltage 55.5 V, Charge end voltage 61.5 V, discharge end voltage 41.0 V, Maximum Charge 80 A, Maximum discharge 300 A, Full cycles 5000
Supercapacitor	**Maxwell BMOD0165 P048 B01** Rate capacitance 165 F, nominal voltage 48 V, Maximum Voltage 51 V, Maximum current 1900 A, Equivalent series resistance initial 6.3 mΩ
*C*_1_, *C*_2_	2.2 mF
*L*_Bo_	10 mH
*L*_Bat_	20 mH
*L*_SC_	20 mH
*L_I_*, *C_I_*, *L_S_*	5 mH, 10 µF, 5 mH

## Nomenclature

$C_{1}$, $C_{2}$	DC link capacitors
$C_{I}$	Inverter output filter capacitors
$D_{PV}$	Boost converter duty cycle
$D_{SC}$	DC/DC supercapacitor converter duty cycle
$D_{bat}$	DC/DC battery converter duty cycle
$L_{Bat}$	DC/DC battery converter inductance
$L_{Bo}$	Boost converter inductance
$L_{I}$	Inverter output filter inductances (inverter side)
$L_{S}$	Inverter output filter inductances (grid side)
$L_{SC}$	DC/DC supercapacitor converter inductance
$P_{Bat}$	Battery power
$P_{PV}$	PV module power
$P_{SC}$	Super-capacitor power
$P_{Stored}$	Total HESS power
$P_{s\;\mathrm{ref}}$	Reference active power set-point
$S_{a}^{+}$; $S_{b}^{+}$; $S_{c}^{+}$; $S_{a}^{-}$; $S_{b}^{-}$; $S_{c}^{-}$	Inverter switching signals
$S_{d}$	Boost converter switching signal
$S_{e}^{+}$; $S_{e}^{-}$	DC/DC battery converter switching signals
$S_{f}^{+}$; $S_{f}^{-}$	DC/DC supercapacitor converter switching signals
$i_{Bat}$	Battery current
$i_{I,a}$; $i_{I,b}$; $i_{I,c}$	Inverter output currents
$i_{PV}$	PV module current
$i_{S,a}$; $i_{S,b}$; $i_{S,c}$	Grid currents
$i_{SC}$	Supercapacitor converter current
$m_{abc}$	Inverter duty cycle
$v_{Bat}$	Battery voltage
$v_{DC\;\mathrm{ref}}$	DC link voltage reference value
$v_{DC}$	DC link voltage
$v_{I,a}$; $v_{I,b}$; $v_{I,c}$	Inverter output voltages
$v_{PV}$	PV module voltage
$v_{S,a}$; $v_{S,b}$; $v_{S,c}$	Grid Voltages
$v_{SC}$	Supercapacitor voltage
τ	Low-pass filter time constant
